# Scaling up a targeted exposome LC-MS/MS biomonitoring method by incorporating veterinary drugs and pesticides

**DOI:** 10.1007/s00216-024-05374-x

**Published:** 2024-06-28

**Authors:** Md Zakir Hossain, Max L. Feuerstein, Yunyun Gu, Benedikt Warth

**Affiliations:** 1 Department of Food Chemistry and Toxicology, Faculty of Chemistry, University of Vienna, Währinger Str. 38, 1090 Vienna, Austria; 2 Exposome Austria, Research Infrastructure and National EIRENE Node, Vienna, Austria; 3 Vienna Doctoral School of Chemistry, University of Vienna, Währinger Straße 42, 1090 Vienna, Austria

**Keywords:** Mass spectrometry, Biomarkers of exposure, Exposome research, Veterinary drugs/antibiotics, Food safety, Xenobiotics

## Abstract

Humans are exposed to a cocktail of food-related and environmental contaminants, potentially contributing to the etiology of chronic diseases. Better characterizing the “exposome” is a challenging task and requires broad human biomonitoring (HBM). Veterinary drugs (VDs)/antibiotics, widely used and regulated in food and animal production, however, are typically not yet included in exposomics workflows. Therefore, in this work, a previously established multianalyte liquid chromatography-tandem mass spectrometry (LC-MS/MS) method covering >80 diverse xenobiotics was expanded by >40 VDs/antibiotics and pesticides. It was investigated if the generic workflow allowed for the successful integration of a high number of new analytes in a proof-of-principle study. The expanded method was successfully in-house validated and specificity, matrix effects, linearity, intra- and inter-day precision, accuracy, limits of quantification, and detection were evaluated. The optimized method demonstrated satisfactory recovery (81–120%) for most of the added analytes with acceptable RSDs (<20%) at three spiking levels. The majority of VDs/antibiotics and pesticides (69%) showed matrix effects within a range of 50–140%. Moreover, sensitivity was excellent with median LODs and LOQs of 0.10 ng/mL and 0.31 ng/mL, respectively. In total, the expanded method can be used to detect and quantify more than 120 highly diverse analytes in a single analytical run. To the best of the authors’ knowledge, this work represents the first targeted biomonitoring method integrating VDs with various other classes of pollutants including plasticizers, PFAS, bisphenols, mycotoxins, and personal care products. It demonstrates the potential to expand targeted multianalyte methods towards additional groups of potentially toxic chemicals.

**Graphical Abstract:**

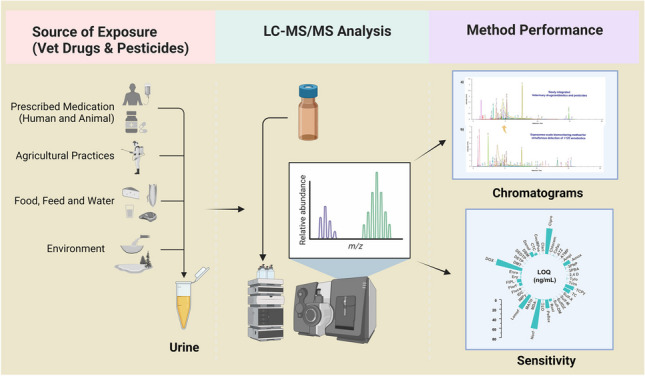

**Supplementary Information:**

The online version contains supplementary material available at 10.1007/s00216-024-05374-x.

## Introduction

Humans are constantly exposed to a diverse mix of environmental chemicals from conception onwards. The human phenotype is a manifestation of the interplay between the genes and the environment, and their interactions are major contributors to chronic diseases [1]. The ability to explore the genetic origins of diseases has been explored previously using large-scale genome-wide association studies (GWAS). Yet, genetics has limited predictive power for the onset of various diseases and can explain only a minority of the total disease risk [2]. Whereas GWAS are widely established, research on environmental determinants for the non-genetic fraction of the total disease risk, i.e., the exposome, is still in its infancy and a solid road map for the implementation of exposome-wide association studies (ExWAS) is still missing. However, recent research indicates that environmental health association factors, e.g., individual diet, smoking, and air pollution, may contribute about 46% of global deaths [1, 3]. The role of specific environmental determinants for many chronic diseases is so far widely unexplored due to difficulties unraveling the link between complex environmental chemical mixtures and disease outcomes. To address an environmental complement to the genome in determining the risk of disease, the “exposome” concept was coined in 2005 and encompasses all environmental, i.e., non-genetic, exposures throughout the lifespan [4]. The individual exposome is extremely dynamic and complex approaches are needed for an accurate characterization resulting in a better understanding of the interplay of environmental factors with biological processes and finally human health. Therefore, the comprehensive study of the individual exposome should ideally incorporate the measurement of environmental influences over the entire life course and associate biological responses to environmental/chemical exposures, diet, behavior, and endogenous processes [5].

Today, millions of organic and inorganic chemicals are listed in the Chemical Abstract Service (CAS) registry, but only as few as 85,000 commercial chemicals are registered under the Toxic Substance Control (TSC) Act of the US Environmental Protection Agency [6] considering their preliminary toxicity information, production volume, and human exposure [7]. The EU-based (HBM4EU, PARC) and US-based (CDC NHANES) programs have already monitored a high number of environmental chemicals and their metabolites, respectively, in a multitude of analytical assays. The full list includes phthalates, phenols, parabens, pesticides, volatile organic compounds (VOCs), polychlorinated biphenyls (PCBs), polycyclic aromatic hydrocarbons (PAHs), and metals in diverse human matrices [8, 9]. Despite these efforts, many chemicals that are listed in the CAS registry or TSC Act are not targeted in most current human biomonitoring (HBM) programs and their health effects remain unexplored. For a better understanding of the so far unknown health risks related to human exposure, more holistic approaches are required to allow a wider coverage of the chemical exposome. Nevertheless, such broad and exposome-scale next-generation HBM methods are still not applied on a routine basis, and the coverage of all “known” exposure chemicals including contaminants of emerging concern and their metabolites is typically limited.

HBM is considered one of the priority areas of the EU Exposure Science Strategy 2020–2030 by developing “The 21^st^-century HBM toolbox” to improve chemical regulations and public health policy in the EU and beyond. Under this framework, HBM can potentially fill remaining research gaps by relating chemical exposure scenarios and disease outcomes [10]. Therefore, further development of novel suitable sample preparation and biomonitoring methods was emphasized to enhance the combined (internal and aggregate) exposure assessment for a wide mixture of environmental chemicals in different target populations and life phases. Targeted mass spectrometry–based analytical methods such as liquid chromatography-tandem mass spectrometry (LC-MS/MS) have been traditionally used to monitor one or several classes of known chemicals in environmental and biological samples resulting in only limited coverage of the exposome [11–14]. Recently, a targeted HBM method for the simultaneous analysis of >80 highly diverse xenobiotic chemicals including plasticizers, perfluorinated alkylated substances, industrial waste products, phyto- and myco-estrogens, food processing products, and personal care products, was developed to circumvent traditional shortcomings of HBM methods and was applied to a cohort of extremely premature infants [15].

**Fig. 1:**
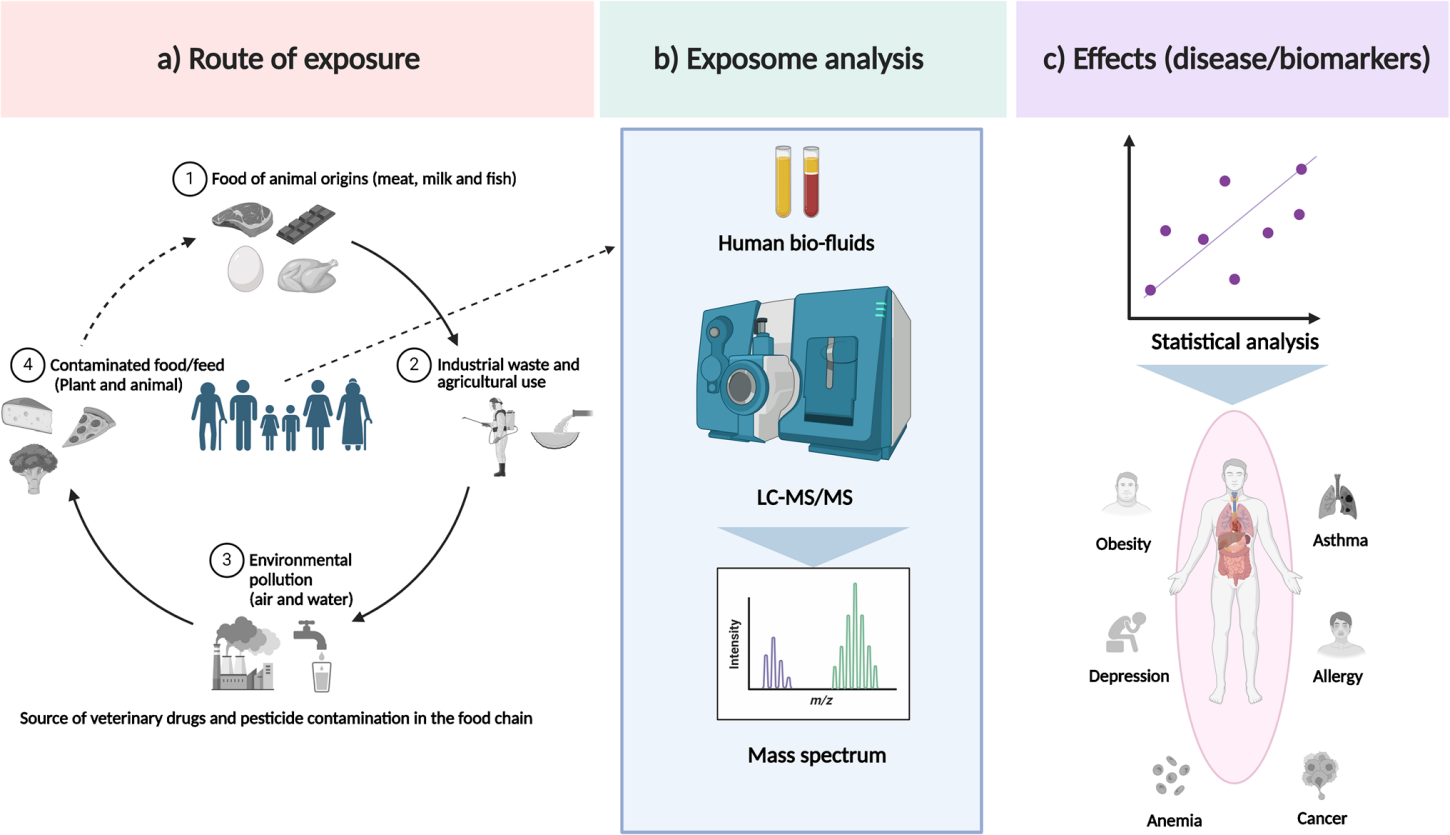
**a** Possible routes for VD/antibiotic and pesticide contamination in the food chain. **b** Schematic workflow for exposome analysis using targeted LC-MS/MS to evaluate biomarkers of xenobiotic exposure in human biofluids and **c** explore the potential association for disease biomarkers and adverse health effects

Veterinary drugs (VDs)/antibiotics are xenobiotic chemicals extensively used to treat or prevent infections and promote growth in the livestock industry. The misuse or excessive use of VDs/antibiotics may result in residues of these drugs in animal-derived food and food products such as meat, milk, and eggs, as well as seafood and honey [16, 17]. VDs/antibiotics are potentially toxic, not only causing antimicrobial resistance but also producing immediate toxicities such as allergic reactions and long-term health effects such as cancer or alteration of human microbiota [17]. Pesticides, another important class of xenobiotic chemicals, are used in agriculture to enhance crop production and control pests including insects, weeds, and fungi. Humans are predominately exposed to pesticides through dietary food intake including food of animal origins, inhalation, and drinking water [18]. The agricultural use of pesticides helps to increase crop production but can result in many unwanted environmental and health-associated short-term and long-term effects including cancer, birth defects, diabetes, reproductive disorders, and Alzheimer’s and Parkinson’s disease [19]. Approximately 1000 pesticides are potentially used on crops and these chemicals and their biomarkers are monitored and regulated by states or international governing organizations, e.g., the EU or US-EPA [6, 20]. To protect humans from undesirable health outcomes, most countries established maximum residue limits (MRLs) of drug residues in foods of animal origin and levels are monitored routinely [21, 22]. Figure 1 presents an overview of potential contamination routes for VDs/antibiotics and pesticides along the human food chain as well as approaches for analyzing the human exposome. This includes the identification of potential risk factors and related biomarkers of biological perturbation and to provide the opportunity to explore underlying health/toxicological effects of xenobiotics in humans. VDs/antibiotics and pesticides enter the human food chain through contaminated food/water from industrial waste, domestic consumption during agricultural farming, and environmental pollution or ingestion of contaminated animal- or plant-based food containing residual VDs or pesticides, either due to administration of VDs/antibiotics and pesticides, or an inappropriate use on agricultural land.

Although there is an increasing number of multianalyte HBM methods reported in recent years, no targeted exposome-scale HBM method thus far included residual veterinary drugs/antibiotics together with pesticides and a multitude of other anthropogenic and natural toxicants in human urine. Therefore, we systematically investigated the potential of a generic targeted HBM method [15] for scaling and integration of new contaminant classes (exemplified here by VDs/antibiotics and pesticides) without affecting the methods’ performance parameters. The expanded method was successfully validated using urine samples as a proof-of-concept sample matrix and is fit-for-purpose to be applied in future large-scale HBM studies to further push the boundaries of our understanding of chemical exposure and its association with human health.

## Materials and methods

### Chemicals

LC-MS grade water, methanol, and acetonitrile (ACN) were obtained from Honeywell (Riedel-del Haen, Germany) and VWR Chemicals (USA). Ammonium fluoride (NH_4_F) was purchased from Honeywell (Fluka, Germany); ammonium acetate (NH_4_Ac) and acetic acid were from Sigma-Aldrich (Darmstadt, Germany). Reference standards for VDs/antibiotics and pesticides with internal standards (ISTDs) were purchased from Sigma-Aldrich and Toronto Research Chemicals (TRC, Canada). In total, 136 authentic analytical standards and 24 isotopically labeled ISTDs were used in this work. A full list of standards and ISTDs is provided in Electronic Supplementary Material (ESM) Table S1.1 and Table S1.2, respectively.

### Preparation of standard solutions

All commercial standards, ISTDs, and stock solutions were stored at −20 °C. Working stock solutions for the VDs/antibiotics and pesticide standards were dissolved in an appropriate pure organic solvent (i.e., LC-MS grade acetonitrile or methanol) to yield a final concentration of mostly 1 mg/mL. In the case of fluoroquinolones (e.g., ciprofloxacin), 50 µL hydrochloric acid (HCl) was added to ensure the solvation of the analytes. The previously used mixture of xenobiotic analytical standards was dissolved in LC-MS grade acetonitrile, methanol, water, or dimethyl sulfoxide to yield a concentration of 1 or 2 mg/mL (according to Jamnik et al. 2022). Detailed information on xenobiotic reference standards is provided in ESM Table S1.2. All 81 xenobiotics from our previously established method were prepared and used according to the validated method published before [15]. The working stock solution containing all xenobiotic analytes including VDs/antibiotics and pesticides (20-fold of the highest calibration level) was prepared in ACN and calibration standards were prepared at eight different concentration levels (see ESM Table S2.1 and Table S2.2). ISTDs were prepared from individual stock solutions at a concentration of 1 mg/mL in an appropriate solvent (ACN or MeOH). For the ISTDs, a working stock solution containing all ISTDs (14 previously used xenobiotics and ten newly added VDs/antibiotics and pesticides) was prepared at 20-fold of the final concentration in ACN. The mix of the 24 ISTDs was then spiked into the samples during the sample preparation (concentration given in ESM Table S3). To improve cost-effectivity, ISTD concentrations were optimized to reduce the necessary amount of standard material, while still ensuring reliable detection and quantification.

### Sample collection and quality controls (QCs)

This research project has been conducted according to all relevant ethical guidelines and was approved by the ethics committee of the University of Vienna (no. 00157). For method validation and matrix-matched calibration, pooled urine was collected from a female volunteer who reduced exposure to xenoestrogen by avoiding foods or cosmetics stored in plastic containers and foods rich in phytoestrogens for 2 days before sample collection. The pooled urine was also used to prepare quality control samples (non-spiked urine “blank” and spiked urine sample “level 3, 10, 30”). Matrix-matched calibration curves were constructed by dissolving the extracted blank urine sample with respective solvent standards after evaporation. All urine samples were stored at −20 °C until the day of use.

### Sample preparation

Sample preparation was performed according to our previously established protocol with slight modifications [15]. In brief, 200 μL of urine samples was mixed with 800 μL of ACN/MeOH (1/1, v/v) containing a mix of 24 ISTDs. Samples were sonicated for 10 min in an ice-cooled water bath and proteins were precipitated during a subsequent freezing step (2 h at −20 °C). The samples were then centrifuged at 4 °C for 10 min at a rate of 18,000×g. The supernatant was collected and dried in a vacuum concentrator at 4 °C. The samples were reconstituted in 200 μL ACN/H_2_O (10/90, v/v), vortexed, and centrifuged for 10 min (18,000×g at 4 °C). The supernatant was transferred to LC vials with inserts and injected into the LC-MS/MS system or stored at −20 °C until the day of analysis. All samples were kept on ice between sample preparation steps.

In addition, urine sample preparation that included β-glucuronidase/sulfatase (*Helix pomatia*) was performed according to the workflow of Fareed et al. [23] for performance comparison purpose. For this, 100 µL urine was spiked at two concentration levels (“level 10” and “level 30”). 2.5 M NH_4_Ac containing β-glucuronidase/arylsulfatase was prepared by dissolving 9.6 g NH_4_Ac in 50 mL water and adding acetic acid until pH 5.5 was reached. The resulting NH_4_Ac buffer (2187 µL) was mixed with β-glucuronidase/arylsulfatase (312 µL) solution (corresponding to 4000 U). A volume of 100 µL of the β-glucuronidase/arylsulfatase enzyme solution in 2.5 M NH_4_Ac buffer was added to the samples and incubated for 16 h at 400 rpm and 37 °C. β-Glucuronidase/sulfatase-treated samples were then extracted as described above with the final reconstitution volume of 100 µL to ensure a consistent dilution factor for better comparability.

### LC-MS/MS instrumentation

LC-MS/MS analysis was performed using a 1290 Infinity II LC system (Agilent) coupled with a Sciex QTrap 6500+ mass spectrometer (Darmstadt, Germany) equipped with a Turbo-V electrospray ionization (ESI) source operated in fast polarity switching mode. Separation of analytes was achieved by a reversed-phase Acquity HSS T3 (1.8 μm, 2.1 × 100 mm) column equipped with a VanGuard (1.8 μm) pre-column from Waters (Vienna, Austria). The LC system comprised a binary pump equipped with a temperature-controlled autosampler and column oven. Autosampler and column oven temperatures were set at 7 °C and 40 °C, respectively. For instrument control, Analyst software (version 1.5.5) was used and SCIEX OS 3.0 used for data analysis.

### LC-MS/MS conditions

The new LC-MS/MS method was based on a modified version of our previously established workflow [15] and incorporated VDs/antibiotics and pesticides. Briefly, 5 µL of the samples were injected into the LC system. The chromatographic separation was performed using a flow rate of 0.4 mL/min and a gradient with water containing 0.3 mM of NH_4_F as eluent A and 98% ACN mixed with 2% of eluent eluent A as eluent B. The gradient elution was based on the method developed before (Jamnik et al., 2022). In brief, eluent B was kept constant at 5.1% between the start and 1 min, increased to 18.4% B between 1 and 1.8 min followed by an increase to 35.7% B until 4.2 min, 49% B until 13 min, before reaching 91.8% B after 14 min. The column was then flushed with 100% B between 14 and 19.5 min and re-equilibrated with 5% B from 19.6 to 20 min. The MS/MS analysis was operated in positive and negative electrospray ionization (ESI) with a scheduled multiple reaction monitoring approach (sMRM) and retention times were optimized individually. For the optimization of the MS/MS parameters for each analyte, single standard solutions with concentrations of 100–1000 ng/mL were injected using a flow injection analysis and declustering potential (DP), collision energy (CE), and collision exit potential (CXP) were optimized to maximize the signal intensity of transitions of parent (Q1) to product ions (Q3).

The final MS conditions were set as follows: ion spray voltage was set to −4500 V in negative mode and to 5500 V in positive mode. Nitrogen was used as collision gas and for all gas flows in the ion source. The CAD gas pressure was set to “medium”, the curtain gas was set to 30 psi, the ion source gas 1 was 80 psi, and the ion source gas 2 was 60 psi. Detailed information on MS and MS/MS optimized parameters for VDs/antibiotics, pesticides, and subset xenobiotics including ISTDs including their polarity, Q1, Q3, DP, CE, and CXP are summarized in ESM Table S4.

### Method validation

The expanded method was validated in-house according to the European Commission (EC, 2002) and Eurachem (2014) guidelines with slight modifications [24, 25]. Method validation was performed for all newly added VDs/antibiotics and pesticides. In addition, a subset of the previously validated xenobiotics was used to compare the new methods’ performance to our previously published work. The validation scheme included the assessment of selectivity, matrix effects, linearity, limit of detection (LOD) and limit of quantification (LOQ), accuracy, and precision (inter- and intra-day). Matrix effects were evaluated in the form of signal suppression or enhancement (SSE) based on the ratios of the slopes of a matrix-matched calibration and a solvent calibration. Matrix effects were considered acceptable if the ratio was in the range of 50–140% [15]. The linearity of the method was investigated based on the calibration curve constructed using mobile phase solutions. For each analyte, the matrix-matched calibration curve was constructed using at least five concentration levels with a coefficient of determination (*R*^2^) limit of > 0.9. A 1/x weighting factor was used for the calibration curve to ensure comparability with the originally published method and to ensure that the accuracy of low-abundance analytes is properly accounted for. Quantification was performed based on peak areas. LODs and LOQs were calculated according to the Eurachem (2014) guideline using the following equations [25]:

${S}_{0}{\prime} =\frac{{S}_{0}}{\surd n}$ where *S*_0_*′* represents the standard deviation for calculating LOD and LOQ, *S*_0_ represents the standard deviation of the calculated concentration of the spiked analyte at level “low,” and *n* is the number of replication injections, and calculated LOD as LOD = 3 x *S*_0_*′*, and LOQ = 10 x *S*_0_*′*.

To assess accuracy and precision, recovery experiments were repeated on three different days and using three different spiking levels (low, medium, and high corresponding to calibration levels 3, 10, and 30 in Supplementary Table 2.2, triplicate extractions per day). Multianalyte standard stocks were spiked into the pooled “blank urine” samples before extraction. The spiking levels were chosen (low and high) according to the previously published data to ensure comparability [15]. The recovery efficiency (R_E_ reported in %) was calculated as concentrations based on the matrix-matched calibration and by comparing calculated concentrations to nominal concentrations. A value for R_E_ of 70–120% was considered acceptable for all analytes. The repeatability (RSD_r_) and intermediate precision (RSD_R_) were assessed as the relative standard deviation of the calculated concentrations in fortified urine samples at the different levels and evaluation was done for results from a single-day (RSD_r_; *n* = 9) or results from 3 days (RSD_R_; *n* = 9). Quality control (QC) samples were measured in triplicate before and after the analysis to ensure stable instrumental performance. For the identification of analytes, four identification parameters were required, namely retention time, presence of quantifier and qualifier ions, and their proper ion ratios.

### Data analysis and software

Data evaluation was performed using the quantitation software “SCIEX OS 3.0” with the “Autopeak” integration algorithm. Integration parameters were optimized individually and results from the automatic peak integration were curated and adjusted manually when needed. Further data calculation was performed using Microsoft Office (Professional Plus 2019). Chemical structures were drawn by ChemDraw 3D 10, chromatograms were extracted using Skyline software (version 22.2) [26], and the figures were created using Inkscape version 0.92.4, OriginPro (2021b), and BioRender (2023).

## Results and discussion

### Analyte selection and chemical coverage

The availability of exposome-scale HBM methods combining a wide range of potential xenobiotic exposures is still limited to date. Methods typically cover biomarkers of a single, or a few, xenobiotic classes often with very similar physicochemical properties or origin [13, 15, 27–29]. Here, we investigate whether an already very broad method can be expanded by additional biomarkers of exposure without modifying the instrumental configuration or interfering with the methods’ performance parameters. This will be of particular importance in the future since many chemicals of emerging concern and so far unknown biotransformation products of already known toxicants are identified currently.

**Fig. 2:**
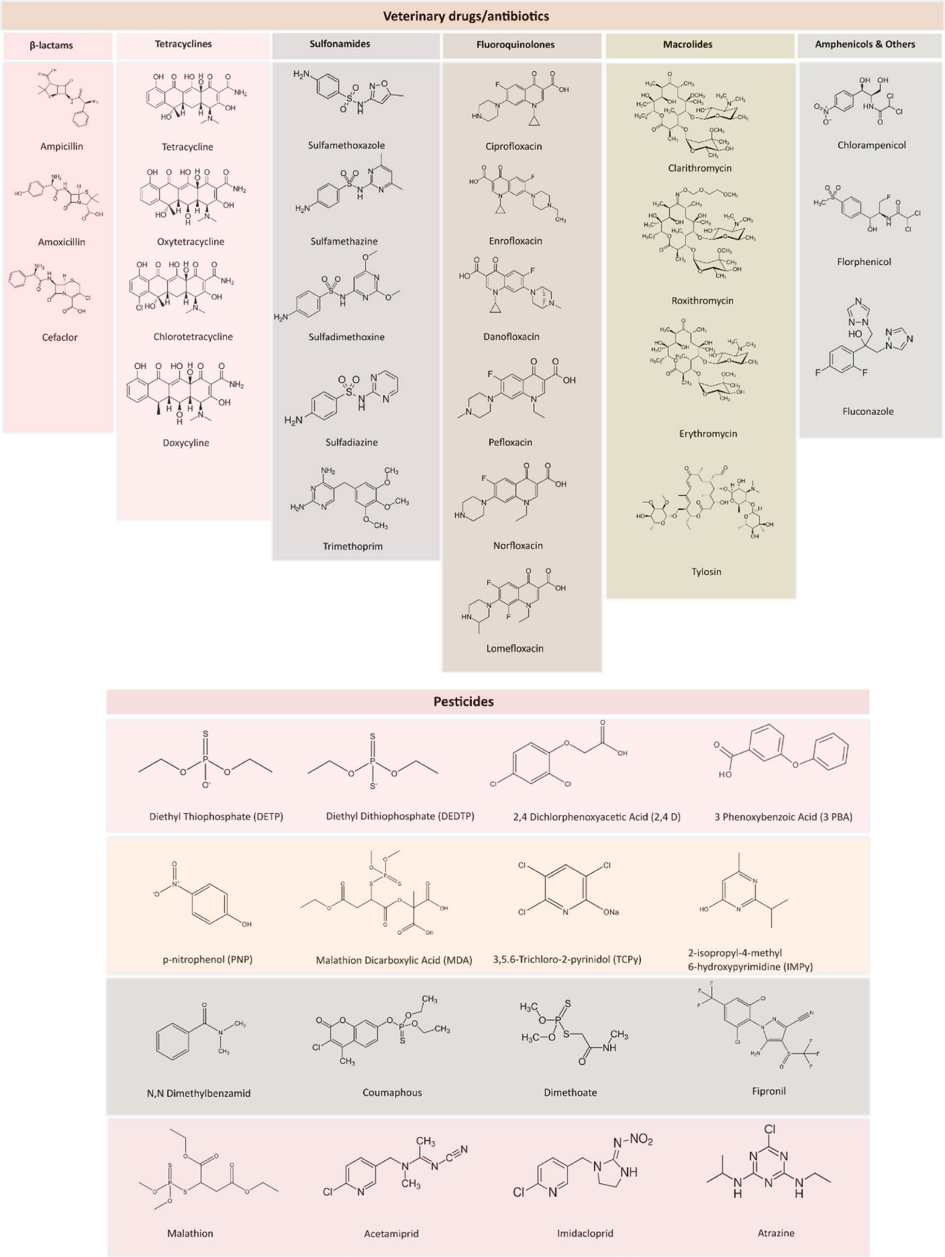
Chemical structures of VDs/antibiotics (β-lactams, tetracyclines, sulfonamides, macrolides, and amphenicols) and pesticides that have been used to demonstrate the scalability of the next-generation biomonitoring approach

To expand and test the scalability of our existing next-generation HBM method, 41 additional analytes, i.e., veterinary antibiotics (*n* = 25) and pesticides and pesticide metabolites (*n* = 16), were selected based on their occurrence data in food, existing biomonitoring data, and their toxicological potential. The five major groups of relevant veterinary antibiotics/drugs include β-lactams, tetracyclines, sulfonamides, fluoroquinolones, and macrolides (Fig. 2). Multiple studies indicated that VDs/antibiotics exposure at residual levels are associated with various adverse health effects such as obesity (e.g., florfenicol, ciprofloxacin, norfloxacin, sulfamethazine, doxycycline), allergic diseases (e.g., sulfamethazine, ciprofloxacin, oxytetracycline), or mental disorders (e.g., ciprofloxacin) [30–33]. Pesticides are an integral part of the chemical exposome and several studies have established a certain correlation between residual pesticide exposure and cancers, diabetes, neurodegenerative disorders (Alzheimer’s and amyotrophic lateral sclerosis), mother and child health outcomes, birth defects, or reproductive diseases [19, 34, 35]. Most of the current LC-MS/MS-based HBM methods are particularly focusing on single pesticide classes and their metabolites in urine samples. We successfully integrate these xenobiotics and several of their metabolites into our HBM method as an additional building-block. The chemical structures of VDs/antibiotics and pesticides added to the method are presented in Fig. 2.

### Optimization of the LC-MS/MS method parameters

**Fig. 3:**
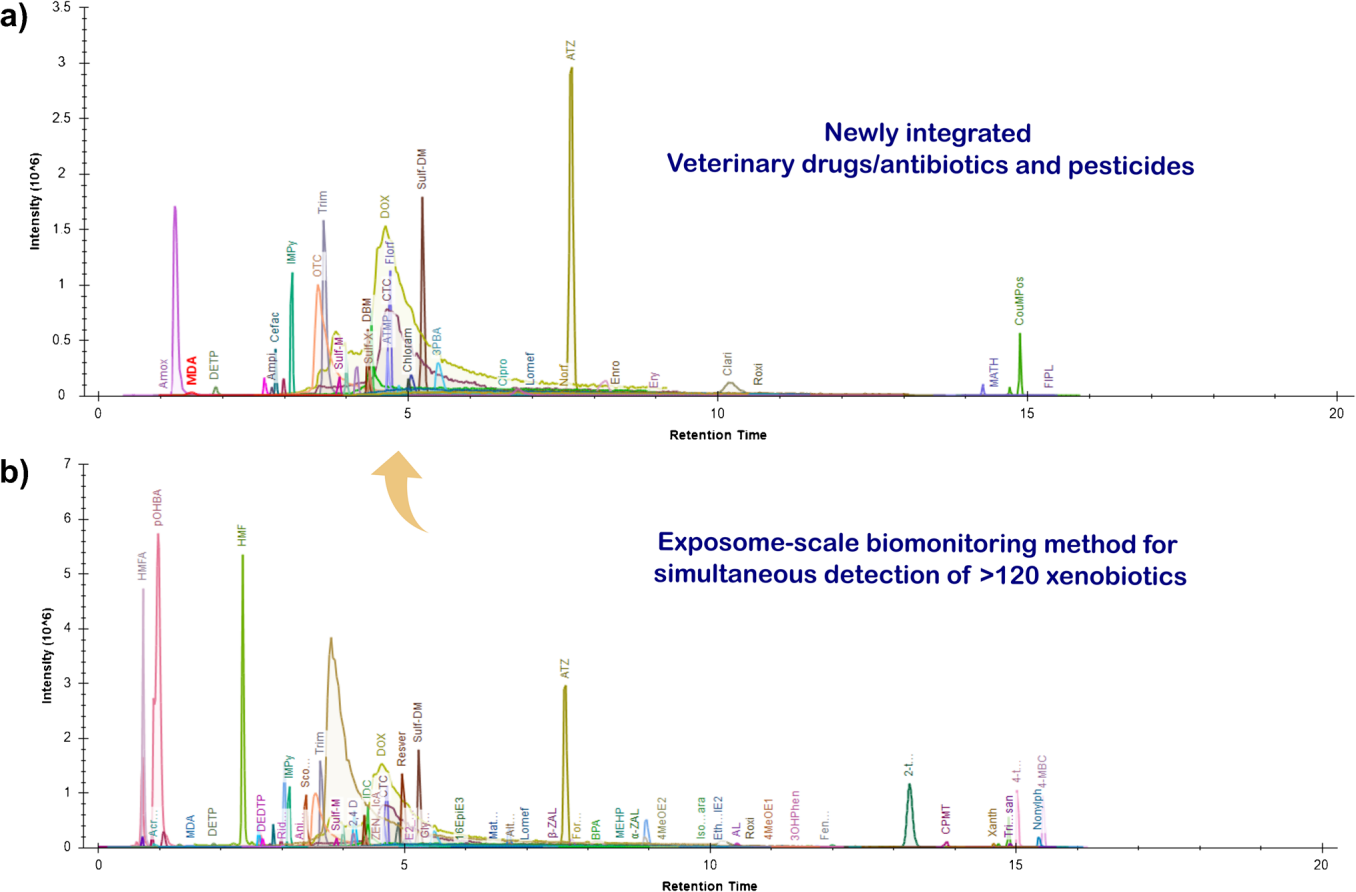
MRM chromatograms of the quantifier transitions for **a** the 41 newly integrated veterinary drugs/antibiotics and pesticides and **b** the final set of all >120 xenobiotics covered by the expanded multi-exposure HBM method. It can be seen that the integration of additional analytes of highly diverse physicochemical properties was feasible, demonstrating the scalability of the next-generation HBM LC-MS/MS method

MS and MS/MS parameters were optimized for each analyte individually in positive and negative ionization mode. For this purpose, direct-flow injections of analytical standards were used and collision energy (CE) and declustering potential (DP) were optimized for the eight most intense transitions of each analyte. Quantifier and qualifier ions were selected based on their sensitivities and signal to noise rations and the finally used transitions are summarized in ESM Table S4 including their mass spectrometric parameters including DP, CE, CXP, RT, and ion species. RT windows for all xenobiotics were tested and optimized using spiked urine samples. The LC parameters have been optimized before for a RP-LC method with a total runtime of 20 min. Analyte separation was favorable for all newly integrated analytes highlighting the generic applicability of the method and its feasibility for integrating additional target substances. The elution times for most analytes were between 2.5 and 15 min. Extracted MRM chromatograms of all analytes from a single analytical run of a calibration standard are reported in Fig. 3. To enhance the analytical sensitivity, NH_4_F was selected as an eluent additive as it has been shown to improve the ionization efficiency for multiple analyte classes such as steroids, bisphenols, or phytoestrogens [13].

### Validation and quality control measures

The expanded LC-MS/MS method was in-house validated by determining selectivity, matrix effects, linearity, accuracy, precision, limit of quantification (LOQ), and limit of detection (LOD) in urine according to the guidelines established by the EC (2002) and Eurochem (2014) with minor modifications [24, 25]. Overall, the validation for VDs/antibiotics and pesticides was successful and the analytical figures of merit are reported in Table 1 and ESM Table S5. To ensure also the constant method performance for the analytes that have been included in the original method after integration of the new analytes, a subset of these analytes was additionally re-evaluated (see Table 1 and ESM Table S5) [15]. The selection covered representatives from various toxicant classes to warrant a comprehensive and broad evaluation.

**Table 1:** Spiking levels used for the in-house validation procedure (*LL*, low level; *ML*, medium level; and *HL*, high level) and extraction recovery (*R*_*E*_) results with repeatability (*RSD*_*r*_) and intermediate precision (*RSD*_*R*_) of the investigated VDs/antibiotics and pesticides

VDs/antibiotics and pesticides	LL/ML/HL (ng/mL)	R_E_ ± RSDR (LL) [%]	R_E_ ± RSDR (ML) [%]	R_E_ ± RSDR (HL) [%]	RSDr (LL / ML / HL) [%]
β-lactams					
Amoxicillin (Amox)	18/60/180	81 ± 9	91 ± 4	85 ± 4	8 / 3 / 4
Ampicillin (Ampi)	0.3/1/3	77 ± 13	96 ± 5	87 ± 5	11 / 5 / 4
Cefaclor (Cefac)	3/10/30	58 ± 9	73 ± 6	66 ± 5	6 / 5 / 4
Tetracyclines					
Chlorotetracycline (CTC)	30/100/300	35 ± 9	49 ± 6	60 ± 4	4 / 3 / 2
Oxytetracycline (OTC)	9/30/90	63 ± 3	78 ± 3	92 ± 2	2 / 3 / 2
Tetracycline (TC)	18/60/180	55 ± 6	76 ± 4	81 ± 19	4 / 3 / 12
Doxycycline (DOX)	18/60/180	76 ± 11	93 ± 7	109 ± 4	9 / 6 / 5
Sulfonamides					
Sulfadimethoxine (Sulf-DM)	0.3/1/3	90 ± 3	106 ± 2	97 ± 2	3 / 2 / 3
Sulfadiazine (Sulf-DZ)	0.09/0.3/0.9	84 ± 12	100 ± 6	91 ± 3	11 / 6 / 3
Sulfamethazine (Sulf-M)	0.09/0.3/0.9	81 ± 10	97 ± 6	90 ± 3	9 / 5 / 3
Sulfamethoxazole (Sulf-X)	0.09/0.3/0.9	85 ± 28	107 ± 4	95 ± 4	25 / 4 / 4
Trimethoprim (Trim)	0.9/3/9	92 ± 2	109 ± 3	98 ± 2	2 / 3 / 2
Fluoroquinolones					
Ciprofloxacin (Cipro)	36/120/360	93 ± 8	108 ± 7	116 ± 9	7 / 7 / 9
Danofloxacin (Danof)	6/20/60	74 ± 10	104 ± 15	107 ± 8	7 / 15 / 8
Enrofloxacin (Enro)	15/50/150	84 ± 5	109 ± 7	107 ± 5	5 / 8 / 5
Lomefloxacin (Lomef)	18/60/180	84 ± 10	107 ± 6	100 ± 4	9 / 7 / 4
Norfloxacin (Norf)	36/120/360	101 ± 8	111 ± 7	110 ± 5	8 / 7 / 6
Pefloxacin (Peflox)	18/60/180	101 ± 5	109 ± 7	103 ± 6	5 / 8 / 6
Macrolides					
Erythromycin A (Ery)	3/10/30	86 ± 6	99 ± 6	94 ± 6	6 / 5 / 6
Roxithromycin (Roxi)	8.1/27/81	106 ± 8	119 ± 3	116 ± 2	9 / 4 / 2
Tylosin (Tylo)	9/30/90	39 ± 19	45 ± 24	44 ± 14	11 / 15 / 3
Clarithromycin (Clari)	2.7/9/27	91 ± 2	101 ± 3	97 ± 2	2 / 3 / 2
Amphenicols and others					
Chloramphenicol (Chloram)	0.9/3/9	97 ± 6	112 ± 4	99 ± 3	6 / 5 / 3
Florfenicol (Florf)	27/90/270	110 ± 7	135 ± 2	114 ± 3	8 / 3 / 3
Fluconazole (Fluco)	0.09/0.3/0.9	96 ± 11	113 ± 5	102 ± 4	11 / 5 / 4
Pesticides					
Acetamiprid (ATMP)	0.015/0.05/0.15	104 ± 4	119 ± 4	107 ± 4	5 / 5 / 4
Atrazine (ATZ)	0.9/3/9	66 ± 6	83 ± 11	75 ± 7	4 / 9 / 5
Dimethoate (DMT)	0.0075/0.025/0.075	96 ± 3	111 ± 4	96 ± 3	3 / 4 / 3
Imidacloprid (IDC)	0.3/1/3	97 ± 6	118 ± 4	104 ± 3	7 / 4 / 3
Malathion (MATH)	0.3/1/3	37 ± 23	47 ± 41	43 ± 29	6 / 9 / 5
Coumaphos (CouMPos)	0.09/0.3/0.9	119 ± 13	155 ± 10	151 ± 9	16 / 14 / 12
Fipronil (FIPL)	9/30/90	71 ± 39	110 ± 13	108 ± 7	32 / 14 / 8
N,N-dimethylbenzamide (DBM)	0.09/0.3/0.9	111 ± 47	70 ± 54	50 ± 35	29 / 23 / 6
Pesticide metabolites					
2-Isopropyl-4-methyl-6-hydroxypyrimidine (IMPy)	0.9/3/9	85 ± 5	101 ± 4	96 ± 3	5 / 4 / 3
3,5,6-Trichloro-2-pyrinidol (TCPy)	30/100/300	66 ± 30	92 ± 11	88 ± 6	23 / 10 / 5
2,4-Dichlorophenoxyacetic acid (2,4 D)	0.9/3/9	90 ± 4	106 ± 3	97 ± 3	4/ 3 / 3
3-Phenoxybenzoic acid (3-PBA)	0.9/3/9	87 ± 4	101 ± 2	94 ± 2	4 / 2 / 2
p-Nitrophenol (PNP)	8.1/27/81	90 ± 5	106 ± 4	96 ± 3	4 / 4 / 3
Diethyl dithiophosphate (DEDTP)	2.7/9/27	81 ± 4	93 ± 4	86 ± 3	4 / 4 / 3
Diethyl thiophosphate (DETP)	0.9/3/9	98 ± 6	112 ± 3	103 ± 3	7 / 3 / 2
Malathion dicarboxylic acid (MDA)	2.7/9/27	101 ± 16	112 ± 13	101 ± 4	16 / 13 / 4

#### Specificity and selectivity

The specificity of the detection method was examined by comparing chromatograms in blank samples and solvent standards. Single chromatographic peaks were observed for all analytes in standard solvent solution without interfering peaks in blank samples. The selectivity was further examined based on the retention time (RT) and peak shape of the target analytes in urine samples compared to those in standard solution. The similarity of RTs and peak shapes of analytes in spiked urine were sufficiently similar to those in pure standard solutions indicating a good method selectivity of method (MRMs of all analytes are presented in Fig. 3).

#### Linearity

To assess the linearity of the new biomonitoring method, at least a five-point calibration curve prepared in pooled urine was generated for all new analytes as well as the subset of previously included xenobiotics. Most analytes (VDs/antibiotics, pesticides, and subset xenobiotics) showed favorable linearity with the coefficient of determination (*R*^2^) values >0.98. Slightly lower *R*^2^ values (0.94–0.97) were reported for chlorotetracycline (CTC), doxycycline (DOX), enrofloxacin (Enro), norfloxacin (Norf), florfenicol (Florf), coumaphos (CouMPos), 3,5,6-trichloro-2-pyrinidol (TCPy), and malathion dicarboxylic acid (MDA; see ESM Table S5).

#### Matrix effects

**Fig. 4:**
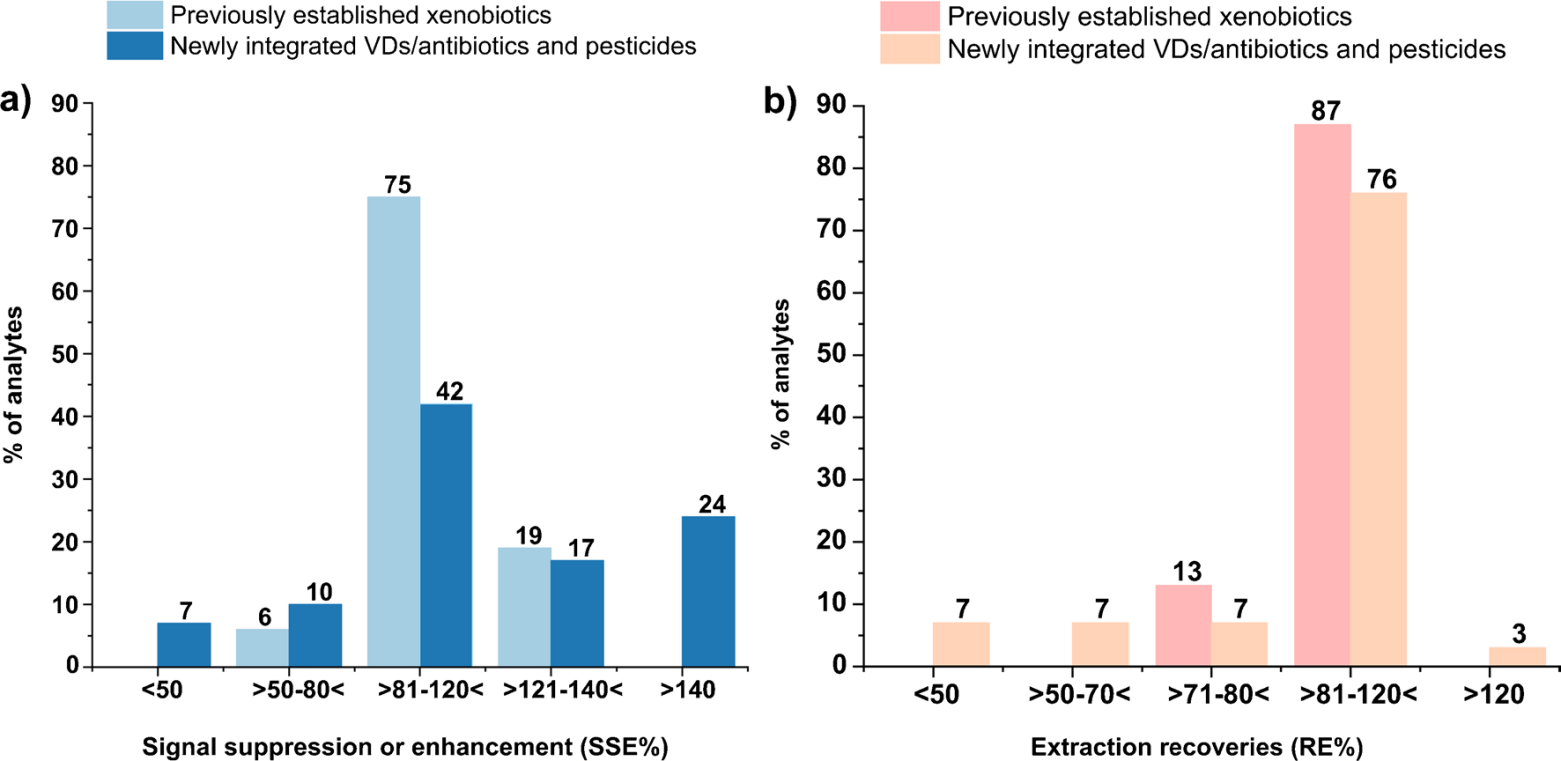
**a** Matrix effects and **b** extraction recovery distribution of the evaluated analytes in urine matrix

Signal suppression or enhancement (SSE) caused by the sample matrix is one of the major challenges in LC-MS and a detailed investigation is necessary when analyzing xenobiotics from different chemical classes in complex biological matrices such as urine. Matrix-induced SSE (%) was calculated as the ratio of the slope of a matrix-matched calibration curve and a calibration in neat solvent. Matrix effects were considered acceptable if the SSE was between 50 and 140% due to the extremely diverse set of analytes. The majority of veterinary and pesticide analytes (69%) showed SSE in this range and the subset of other xenobiotics [15] showed matrix effects within the range from 71 to 128% (Fig. 4). However, strong matrix effects have also been observed for a few VDs/antibiotics and pesticides with SSE % values higher than 150% or smaller than 50%. DOX, danofloxacin (Danof), Enro, lomefloxacin (Lomef), Norf, pefloxacin (Peflox), erythromycin (Ery), and diethyl dithiophosphate (DEDTP) showed substantial signal enhancement (>150%), whereas amoxicillin (Amox), tetracycline (TC), fluconazole (Fluco), and 2-isopropyl-4-methyl-6-hydroxypyrimidine (IMPy) displayed substantial ion suppression (<50%) as shown in ESM Table S5. Similar findings were previously reported for VDs/antibiotics where strong matrix effects (>130% or <70%) have been observed for most analytes in urine [36]. Matrix-matched calibration curves were used for the quantification to account for signal suppression or enhancement in this work.

#### Recovery, accuracy, and precision

The extraction efficiency of the analytical method was considered excellent if the recovery rate of the target analyte was near or equal to 100%. Analyte recovery and relative standard deviation (RSD) between replicate extractions were used to determine accuracy and precision in bioanalytical methods. In general, recovery rates of 50–120% and an RSD <20% were considered acceptable ranges. Due to a lack of a suitable certified reference material covering the target analytes, spiking experiments in triplicates at three different concentrations were performed to evaluate recovery and precision. The spiking levels were generally selected as follows: *low level* (*LL*): 3× the approximated LOQ (limit of quantification was calculated according to signal/noise ratio of 10 in preliminary experiments), *medium level* (*ML*): 10× the LOQ, and *high level* (*HL*): 30× the LOQ in pooled urine (Table 1). Fortified samples were extracted using the same sample preparation steps as mentioned in the section above. The recovery rate for VDs/antibiotics and pesticides ranged between 81 and 120% for 76% of the newly integrated analytes as shown in Fig. 4b. The β-lactam cefaclor (Cefac) and the tetracycline CTC showed lower recovery. All macrolide antibiotics except Tylo showed excellent recoveries (86–106%) with the combination of acetonitrile: methanol (1:1, v/v) as extraction solvent which is in agreement with work published elsewhere [37]. In addition, results from the re-evaluation of the subset of previously included xenobiotics (*n* = 16) were compared to the reported results of the original method [15]. The recoveries for the three fortification levels are presented in detail in Table 1. These results were comparable as shown in ESM Table S7 where the subset of re-evaluated xenobiotics showed 71–128% recovery rate which was also in line with other published work [27, 28]. Satisfactory recoveries (LL, 66–104%; ML, 83–119%; and HL, 75–107%) were also achieved for pesticides except malathion (MATH; 37–47%) and CouMPos (119–155%), which is comparable to results from a recently published method for the analysis of pesticides in urine [38].

Good precision (intermediate precision) was achieved for most VDs/antibiotics and pesticide analytes at LL, ML, and HL with RSDR <20% except for tylosin (Tylo; 24% at ML), sulfamethoxazole (Sulf-X; 28% at LL), fipronil (FIPL; 39% at LL), TCPy (30% at LL), and all three levels (LL, ML, and HL) for MATH and N,N-dimethylbenzamide (DBM). Moreover, repeatability (intra-day precision, RSDr) was assessed as three replicates of each of three separate spiked urines. Most analytes showed excellent RSDs <20% except for the pesticides, TCPy (23% at LL), Sulf-X (25% at LL), and all three levels for MATH and DBM. For the re-evaluated subset of xenobiotics, RSDr was <20% for all analytes.

#### Sensitivity (LOD and LOQ)

**Fig. 5:**
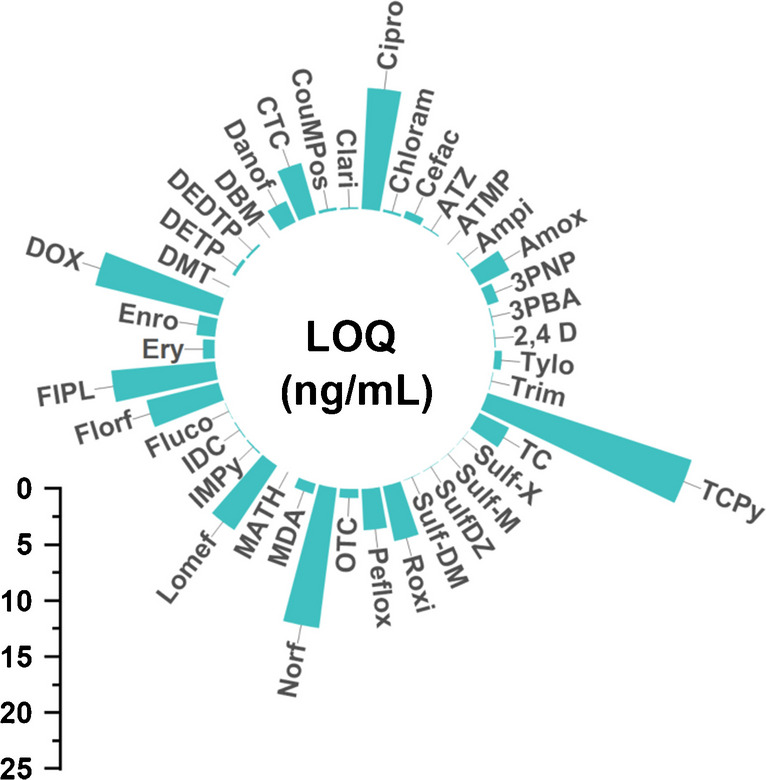
Limits of quantification of veterinary drugs/antibiotics and pesticides as determined during method validation

Estimation of LOD and LOQ values was performed according to the Eurachem (2014) guidelines as described above. The median LOD and LOQ for VDs/antibiotics and pesticides are 0.10 ng/mL (ranging from 0.0003 to 6.3 ng/mL) and 0.31 ng/mL (0.0008–19 ng/mL), respectively, as shown in Fig. 5. The LOQs and LODs for VDs/antibiotics and pesticides can be reviewed in more detail in ESM Table S5. Nevertheless, the LOQs for the fluoroquinolones ciprofloxacin (Cipro), Danof, Enro, Lomef, Norf, and Peflox were comparably high (1.7–13 ng/mL). Similarly, LOQs of the tetracycline TC and DOX and the amphenicol Florf (>3 ng/mL), as well as the pesticide TCPy (19 ng/mL) were higher than LOQs of most other investigated analytes. For the subset of the previously validated xenobiotics (*n* = 16), the median LOD and LOQ were 0.0023 ng/mL (0.0001–0.052 ng/mL) and 0.023 ng/mL (0.0007–0.52 ng/mL), respectively. The LOQ values are highly comparable with the previously reported LOQs for the same analytes and highlights the excellent robustness of the original method [15] and its scalability. A comparison of a S/N-based approach to calculate LODs and LOQs to results from the standard deviation–based approach (based on spiked urine samples) used in this work was performed to ensure comparability of the reported data and can be reviewed in more detail in ESM Table S6.

#### β-Glucuronidase-treated pooled urine samples

**Fig. 6:**
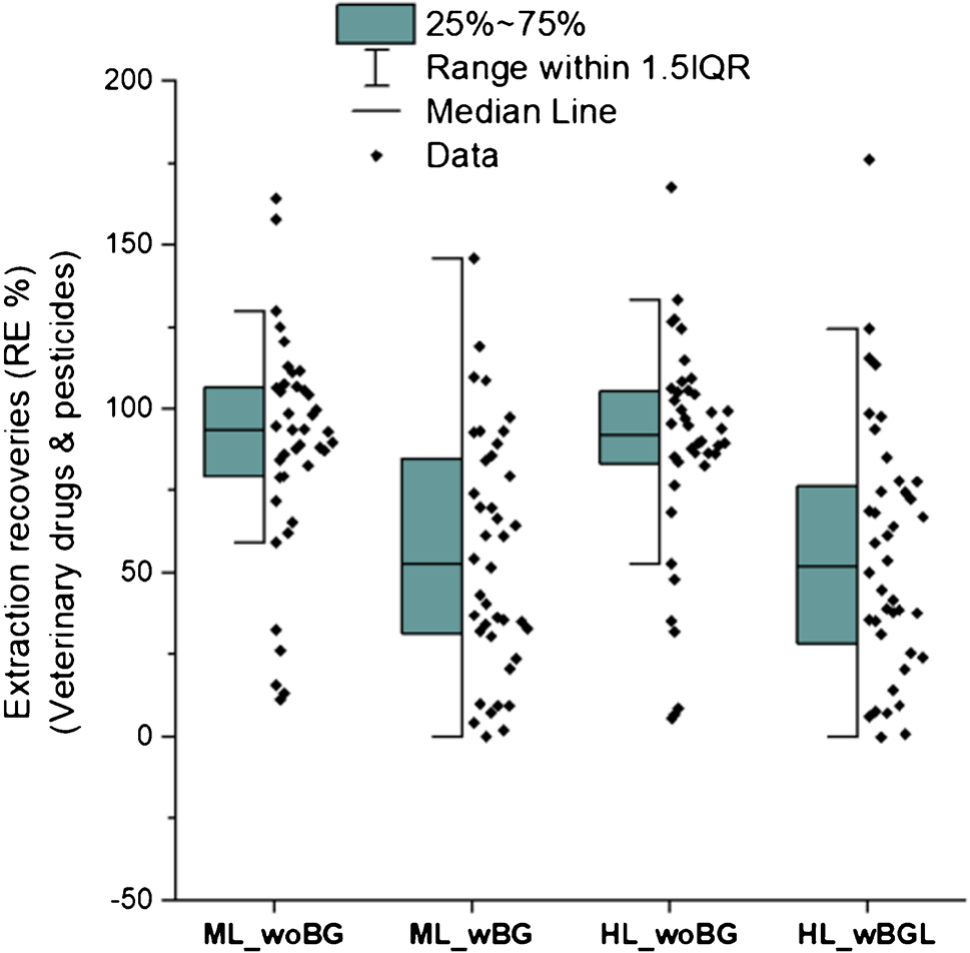
Extraction recoveries (R_E_%) of β-glucuronidase-treated and non-treated pooled urine samples at two spiking levels (ML, medium level; HL, high level). The calculations were based on matrix-matched calibration of at least five concentration levels. woBG, without β-glucuronidase; wBG, with β-glucuronidase

The human body often eliminates xenobiotics through xenobiotic metabolism with phase I activation (oxidation, reduction, and hydrolysis), phase II conjugation (sulfation, acetylation, glucuronidation, and glutathione), and excretion of xenobiotics via bile or urine. A recent deconjugation study concluded that β-glucuronidase from *H. pomatia* is the most efficient enzyme for deconjugation of phase II metabolites to capture the total exposure in urine samples [23]. Among different hydrolysis enzymes, the *H. pomatia* enzyme was selected to investigate deconjugation in an additional experiement as it shows better hydrolysis efficiencies of xenobiotics in the urine despite multi-analyte contamination due to its crude nature [23]. Pooled urine was fortified at two spiking levels and lower extraction efficiencies were observed compared to the non-*β-glucuronidase-treated* urine samples (Fig. 6). The reason for lower extraction recoveries might be the fact that the matrix has a higher background as similar findings were reported by Fareed et al. [23]. For details, see the ESM Table S8.

### Comparison with existing multi-analyte assays and future application potential

This work represents the further evolution of our originally developed highly generic LC-MS/MS method to determine xenoestrogens and other toxicants and endogenous hormones in biological matrices [13], which was further expanded to a final coverage of 81 diverse xenobiotics and biomarkers of chemical exposure [15]. Here, we demonstrated the feasibility to include a large number of additional exposure chemicals without losing analytical performance. Another targeted LC-MS/MS method has been validated for 121 environmental chemicals from seven chemical classes, particularly focusing on plasticizers, phenols, and pesticides [28], and recently, a targeted LC-MS/MS method has been validated for the detection of 50 biomarkers of common environmental exposures in urine samples [27]. However, full incorporation of various analyte classes including VDs/antibiotics and pesticides is typically limited and only a few chemical classes are covered by such methods.

Technical developments in the field of high-resolution mass spectrometry (HRMS) led to a growing number of HRMS-based screening methods published in recent years [39]. Although such approaches likely cover a more exhaustive chemical space, they currently cannot offer the sensitivity required for monitoring a high number of environmental contaminants at ultra-trace levels [40]. This is of special relevance when tackling the chronic low-dose exposures that may cause chronic diseases in human populations. Therefore, fully quantitative targeted multi-analyte methods can be regarded as the current gold standard to assess various exposures due to their better sensitivity and robustness.

To the best of our knowledge, this is the first exposome-scale urinary HBM study to determine more than 120 xenobiotics from multiple chemical classes and allow the integrated assessment of VDs/antibiotics and pesticides together with a broad panel of other exposure chemicals in a single LC-MS/MS run. It would be beneficial to verify the performance parameters established after enzymatic hydrolysis using beta-glucuronidase**/**arylsulfatase treatment further, although this sample pre-treatment often introduces interferences [23]. Either with or without enzymatic deconjugation, this approach holds the potential to being applied in large-scale ExWAS for investigating the role of environmental and food contaminants, including veterinary drugs/antibiotics, in human health and disease.

## Conclusion

The scalability of an exposome-scale human biomonitoring method using LC-MS/MS was demonstrated by expanding the panel of target analytes to more than 120 highly diverse xenobiotics including several commonly used VDs/antibiotics, pesticides as well as various other toxicants including plasticizers, personal care products, industrial chemicals, mycotoxins, phytoestrogens, phytotoxins, or PFAS for the first time. The specificity, linearity, matrix effects, accuracy, and precision were evaluated and the expanded method was mostly successfully validated according to established guidelines. This updated biomonitoring method can be applied for the analysis of a wide range of chemical exposures in urine samples on a routine basis. The method includes a uniquely diverse panel of multiple chemical classes, it offers the necessary coverage and sensitivity for exposome-scale HBM and is suitable for large cohort population studies. Moreover, the method has the potential to be further expanded in the future as successfully exemplified in this work. Given the broad range of physicochemical properties of the included analytes compromises in method performance are unavoidable, however, even the semi-quantitative assessment of certain analytes can be of value. The large-scale, comprehensive and (semi-) quantitative assessment of real-life exposure scenarios for broad analyte panels are of great value for interdisciplinary research domains ranging from epidemiology to precision medicine as well as for policymakers in order to improve public health. The routine application of our new and expanded HBM method in large-scale HBM studies will further improve our understanding of chemical co-exposure and toxicological mixture effects. Considering the complexity of the topic, monitoring of these >120 xenobiotics including integrated VDs/antibiotics and pesticides can be regarded as extremely useful to identify exposure sources, assess related health effects, and ultimately, improve human health.

### Supplementary Information

Below is the link to the electronic supplementary material.

Supplementary file1 (XLSX 66.5 KB)

## References

[1] Chung MK, Rappaport SM, Wheelock CE, Nguyen VK, Van Der Meer TP, Miller GW, Vermeulen R, Patel CJ. Utilizing a biology-driven approach to map the exposome in health and disease: an essential investment to drive the next generation of environmental discovery. Environ Health Perspect. 2021;129: 085001. 10.1289/EHP8327. 34435882 10.1289/EHP8327 PMC8388254

[2] Vermeulen R, Schymanski EL, Barabási A-L, Miller GW. The exposome and health: where chemistry meets biology. Science. 2020;367:392–6. 10.1126/science.aay3164. 31974245 10.1126/science.aay3164 PMC7227413

[3] Rappaport SM. Genetic factors are not the major causes of chronic diseases. PLOS ONE. 2016;11: e0154387. 10.1371/journal.pone.0154387. 27105432 10.1371/journal.pone.0154387 PMC4841510

[4] Wild CP. Complementing the genome with an “exposome”: the outstanding challenge of environmental exposure measurement in molecular epidemiology. Cancer Epidemiol Biomarkers Prev. 2005;14:1847–50. 10.1158/1055-9965.EPI-05-0456. 16103423 10.1158/1055-9965.EPI-05-0456

[5] Miller GW, Jones DP. The nature of nurture: refining the definition of the exposome. Toxicol Sci. 2014;137:1–2. 10.1093/toxsci/kft251. 24213143 10.1093/toxsci/kft251 PMC3871934

[6] US-EPA (2016) Tolerances and exemptions for pesticide chemical residues in food. (40 CFR 180), https://www.govinfo.gov/app/details/CFR-2016-title40-vol26/CFR-2016-title40-vol26-part180, Accessed on 17/10/2023.

[7] Krimsky S. The unsteady state and inertia of chemical regulation under the US Toxic Substances Control Act. PLOS Biol. 2017;15: e2002404. 10.1371/journal.pbio.2002404. 29252997 10.1371/journal.pbio.2002404 PMC5734679

[8] CDC (2023) Center for Disease Control and Prevention-National Biomonitoring Program. https://www.cdc.gov/biomonitoring/environmental_chemicals.html, Accessed on 17/10/2023.

[9] HBM4EU (2022) Human Biomonitoring Project, Grant Agreement No. 733032. https://www.hbm4eu.eu/, Accessed on 17/10/2024.

[10] Zare Jeddi M, Hopf NB, Louro H, Viegas S, Galea KS, Pasanen-Kase R, Santonen T, Mustieles V, Fernandez MF, Verhagen H, Bopp SK, Antignac JP, David A, Mol H, Barouki R, Audouze K, Duca R-C, Fantke P, Scheepers P, Ghosh M, Van Nieuwenhuyse A, Lobo Vicente J, Trier X, Rambaud L, Fillol C, Denys S, Conrad A, Kolossa-Gehring M, Paini A, Arnot J, Schulze F, Jones K, Sepai O, Ali I, Brennan L, Benfenati E, Cubadda F, Mantovani A, Bartonova A, Connolly A, Slobodnik J, Bruinen de Bruin Y, van Klaveren J, Palmen N, Dirven H, Husøy T, Thomsen C, Virgolino A, Röösli M, Gant T, von Goetz N, Bessems J. Developing human biomonitoring as a 21st century toolbox within the European exposure science strategy 2020–2030. Environ Int. 2022;168: 107476. 10.1016/j.envint.2022.107476. 36067553 10.1016/j.envint.2022.107476

[11] Asimakopoulos AG, Xue J, De Carvalho BP, Iyer A, Abualnaja KO, Yaghmoor SS, Kumosani TA, Kannan K. Urinary biomarkers of exposure to 57 xenobiotics and its association with oxidative stress in a population in Jeddah, Saudi Arabia. Environ Res. 2016;150:573–81. 10.1016/j.envres.2015.11.029. 26654562 10.1016/j.envres.2015.11.029

[12] Braun D, Ezekiel CN, Abia WA, Wisgrill L, Degen GH, Turner PC, Marko D, Warth B. Monitoring early life mycotoxin exposures via LC-MS/MS breast milk analysis. Anal Chem. 2018;90:14569–77. 10.1021/acs.analchem.8b04576. 30449087 10.1021/acs.analchem.8b04576

[13] Preindl K, Braun D, Aichinger G, Sieri S, Fang M, Marko D, Warth B. A generic liquid chromatography−tandem mass spectrometry exposome method for the determination of xenoestrogens in biological matrices. Anal Chem. 2019;91:11334–42. 10.1021/acs.analchem.9b02446. 31398002 10.1021/acs.analchem.9b02446

[14] Xue J, Lai Y, Liu C-W, Ru H. Towards mass spectrometry-based chemical exposome: current approaches, challenges, and future directions. Toxics. 2019;7:41. 10.3390/toxics7030041. 31426576 10.3390/toxics7030041 PMC6789759

[15] Jamnik T, Flasch M, Braun D, Fareed Y, Wasinger D, Seki D, Berry D, Berger A, Wisgrill L, Warth B. Next-generation biomonitoring of the early-life chemical exposome in neonatal and infant development. Nat Commun. 2022;13:2653. 10.1038/s41467-022-30204-y. 35550507 10.1038/s41467-022-30204-y PMC9098442

[16] Baynes RE, Dedonder K, Kissell L, Mzyk D, Marmulak T, Smith G, Tell L, Gehring R, Davis J, Riviere JE. Health concerns and management of select veterinary drug residues. Food Chem Toxicol. 2016;88:112–22. 10.1016/j.fct.2015.12.020. 26751035 10.1016/j.fct.2015.12.020

[17] Bedale WA. Veterinary drug residues in foods of animal origin. In: Smulders FJM, Rietjens IMCM, Rose M (eds) Chemical hazards in foods of animal origin. Brill | Wageningen Academic, 2019;pp 51–79.

[18] Margni M, Rossier D, Crettaz P, Jolliet O. Life cycle impact assessment of pesticides on human health and ecosystems. Agric Ecosyst Environ. 2002;93:379–92. 10.1016/S0167-8809(01)00336-X. 10.1016/S0167-8809(01)00336-X

[19] Mostafalou S, Abdollahi M. Pesticides and human chronic diseases: evidences, mechanisms, and perspectives. Toxicol Appl Pharmacol. 2013;268:157–77. 10.1016/j.taap.2013.01.025. 23402800 10.1016/j.taap.2013.01.025

[20] Regulation (EC) No 396/2005 of the European Parliament and of the Council of 23 February 2005 on maximum residue levels of pesticides in or on food and feed of plant and animal origin and amending Council Directive 91/414/EEC. Off J L. 2005;70:1–16.

[21] Commission Regulation (EU) No 37/2010 of 22 December 2009 on pharmacologically active substances and their classification regarding maximum residue limits in foodstuffs of animal origin (Text with EEA relevance). Off J L. 2010;15:1–72.

[22] FAO. Maximum residue limits (MRLS) and risk management recommendations (RMRS) for residues of veterinary drugs in food 2021.

[23] Fareed Y, Braun D, Flasch M, Globisch D, Warth B. A broad, exposome-type evaluation of xenobiotic phase II biotransformation in human biofluids by LC-MS/MS. Exposome. 2022;2:osac008. 10.1093/exposome/osac008. 10.1093/exposome/osac008

[24] EC (2002) 2002/657/EC: Commission Decision of 12 August 2002 implementing Council Directive 96/23/EC concerning the performance of analytical methods and the interpretation of results (text with EEA relevance) (notified under document number C(2002) 3044).

[25] Eurachem (2014) The fitness for purpose of analytical methods-a laboratory guide to method validation and related topics, 2nd edition. 2014.

[26] Skyline (2023) Skyline for small molecules, https://skyline.ms/project/home/begin.view, Accessed on 27/12/2024.

[27] Jagani R, Pulivarthi D, Patel D, Wright RJ, Wright RO, Arora M, Wolff MS, Andra SS. Validated single urinary assay designed for exposomic multi-class biomarkers of common environmental exposures. Anal Bioanal Chem. 2022;414:5943–66. 10.1007/s00216-022-04159-4. 35754089 10.1007/s00216-022-04159-4 PMC9326253

[28] Zhu H, Chinthakindi S, Kannan K. A method for the analysis of 121 multi-class environmental chemicals in urine by high-performance liquid chromatography-tandem mass spectrometry. J Chromatogr A. 2021;1646: 462146. 10.1016/j.chroma.2021.462146. 33895641 10.1016/j.chroma.2021.462146 PMC8106663

[29] Yao Y, Shao Y, Zhan M, Zou X, Qu W, Zhou Y. Rapid and sensitive determination of nine bisphenol analogues, three amphenicol antibiotics, and six phthalate metabolites in human urine samples using UHPLC-MS/MS. Anal Bioanal Chem. 2018;410:3871–83. 10.1007/s00216-018-1062-2. 29671029 10.1007/s00216-018-1062-2

[30] Hu Y, Zhu Q, Wang Y, Liao C, Jiang G. A short review of human exposure to antibiotics based on urinary biomonitoring. Sci Total Environ. 2022;830: 154775. 10.1016/j.scitotenv.2022.154775. 35339554 10.1016/j.scitotenv.2022.154775

[31] Wang Q, Duan YJ, Wang SP, Wang LT, Hou ZL, Cui YX, Hou J, Das R, Mao DQ, Luo Y. Occurrence and distribution of clinical and veterinary antibiotics in the faeces of a Chinese population. J Hazard Mater. 2020;383: 121129. 10.1016/j.jhazmat.2019.121129. 31546217 10.1016/j.jhazmat.2019.121129

[32] Zhang W-X, Zeng X-X, Chen Q, Yu K, Zheng H, Yu X-G, Zhang Y-J, Zhang J, Huang H-Y, Huang L-S. Prenatal environmental antibiotics and fetal and postnatal growth: a biomonitoring-based prospective study in Eastern China. Chemosphere. 2022;288: 132657. 10.1016/j.chemosphere.2021.132657. 34699881 10.1016/j.chemosphere.2021.132657

[33] Zhou X, Cuasquer GJP, Li Z, Mang HP, Lv Y. Occurrence of typical antibiotics, representative antibiotic-resistant bacteria, and genes in fresh and stored source-separated human urine. Env Int. 2021;146: 106280. 10.1016/j.envint.2020.106280. 33395931 10.1016/j.envint.2020.106280 PMC7786438

[34] Ntzani EE, Ntritsos G CM, Evangelou E, Tzoulaki I. Literature review on epidemiological studies linking exposure to pesticides and health effects. EFSA Support Publ 2013;10. 10.2903/sp.efsa.2013.EN-497.

[35] Yusa V, Millet M, Coscolla C, Roca M. Analytical methods for human biomonitoring of pesticides A review. Anal Chim Acta. 2015;891:15–31. 10.1016/j.aca.2015.05.032. 26388361 10.1016/j.aca.2015.05.032

[36] Huang W, Qiu Q, Chen M, Shi J, Huang X, Kong Q, Long D, Chen Z, Yan S. Determination of 18 antibiotics in urine using LC-QqQ-MS/MS. J Chromatogr B. 2019;1105:176–83. 10.1016/j.jchromb.2018.12.019. 10.1016/j.jchromb.2018.12.019 30597417

[37] Tao Y, Yu G, Chen D, Pan Y, Liu Z, Wei H, Peng D, Huang L, Wang Y, Yuan Z. Determination of 17 macrolide antibiotics and avermectins residues in meat with accelerated solvent extraction by liquid chromatography–tandem mass spectrometry. J Chromatogr B. 2012;897:64–71. 10.1016/j.jchromb.2012.04.011. 10.1016/j.jchromb.2012.04.011 22542398

[38] Fišerová PS, Kohoutek J, Degrendele C, Dalvie MA, Klánová J. New sample preparation method to analyse 15 specific and non-specific pesticide metabolites in human urine using LC-MS/MS. J Chromatogr B. 2021;1166:122542. 10.1016/j.jchromb.2021.122542. 10.1016/j.jchromb.2021.122542 33540146

[39] Oesterle I, Braun D, Berry D, Wisgrill L, Rompel A, Warth B. Polyphenol exposure, metabolism, and analysis: a global exposomics perspective. Annu Rev Food Sci Technol. 2021;12:461–84. 10.1146/annurev-food-062220-090807. 33351643 10.1146/annurev-food-062220-090807

[40] Flasch M, Koellensperger G, Warth B. Comparing the sensitivity of a low- and a high-resolution mass spectrometry approach for xenobiotic trace analysis: an exposome-type case study. Anal Chim Acta. 2023;1279:341740. 10.1016/j.aca.2023.341740. 37827628 10.1016/j.aca.2023.341740

